# Antiviral Activity of Uridine Derivatives of 2-Deoxy Sugars against Tick-Borne Encephalitis Virus

**DOI:** 10.3390/molecules24061129

**Published:** 2019-03-21

**Authors:** Ewelina Krol, Ilona Wandzik, Gabriela Brzuska, Luděk Eyer, Daniel Růžek, Boguslaw Szewczyk

**Affiliations:** 1 Department of Recombinant Vaccines, Intercollegiate Faculty of Biotechnology, University of Gdansk and Medical University of Gdansk, Abrahama 58, 80-307 Gdansk, Poland; gabriela.brzuska@phdstud.ug.edu.pl (G.B.); szewczyk@biotech.ug.gda.pl (B.S.); 2 Department of Organic Chemistry, Bioorganic Chemistry and Biotechnology, Faculty of Chemistry, Silesian University of Technology, Krzywoustego 4, 44-100 Gliwice, Poland; Ilona.Wandzik@polsl.pl; 3 Biotechnology Center, Silesian University of Technology, Krzywoustego 8, 44-100 Gliwice, Poland; 4 Department of Virology, Veterinary Research Institute, Hudcova 70, CZ-62100 Brno, Czech Republic; eyer@vri.cz (L.E.); ruzekd@paru.cas.cz (D.R.); 5 Institute of Parasitology, Biology Centre of the Czech Academy of Sciences, Branisovska 31, CZ-37005 Ceske Budejovice, Czech Republic

**Keywords:** tick-borne encephalitis, antivirals, glycosylation inhibition, uridine, 2-deoxy sugars

## Abstract

Tick-borne encephalitis virus (TBEV) is a causative agent of tick-borne encephalitis (TBE), one of the most important human infections involving the central nervous system. Although effective vaccines are available on the market, they are recommended only in endemic areas. Despite many attempts, there are still no specific antiviral therapies for TBEV treatment. Previously, we synthesized a series of uridine derivatives of 2-deoxy sugars and proved that some compounds show antiviral activity against viruses from the Flaviviridae and Orthomyxoviridae families targeting the late steps of the *N*-glycosylation process, affecting the maturation of viral proteins. In this study, we evaluated a series of uridine derivatives of 2-deoxy sugars for their antiviral properties against two strains of the tick-borne encephalitis virus; the highly virulent TBEV strain Hypr and the less virulent strain Neudoerfl. Four compounds (**2**, **4**, **10**, and **11**) showed significant anti-TBEV activity with IC_50_ values ranging from 1.4 to 10.2 µM and low cytotoxicity. The obtained results indicate that glycosylation inhibitors, which may interact with glycosylated membrane TBEV E and prM proteins, might be promising candidates for future antiviral therapies against TBEV.

## 1. Introduction

Serious viral infectious diseases transmitted by invertebrate vectors have always been a major public health problem in all parts of the world. One of the diseases of growing importance which belongs to this group is tick-borne encephalitis (TBE). TBE is a seasonal disorder of the central nervous system which may lead to serious medical complications in humans, including meningitis, meningoencephalitis, or even death [1]. The causative agent of the zoonosis-tick-borne encephalitis virus (TBEV) is transmitted, as the name implies, by ticks. TBEV is a member of the *Flavivirus* genus in the Flaviviridae family which includes, among others, hepatitis C virus (HCV), West Nile virus, Zika virus, dengue virus, Japanese encephalitis virus, and yellow fever virus. 

TBEV is a small, single-stranded, positive-polarity RNA virus with an enveloped virion approximately 50 nm in diameter [2]. Three subtypes of TBEV, including European (TBEV-Eu), Siberian (TBEV-Sib), and Far Eastern (TBEV-FE), are known [3], but a new subtype derived from TBEV-Sib, Baikalian (TBEV-Bkl), has also been described [4]. Recently, another subtype, Himalayan (Him-TBEV), was identified in wild rodents [5]. The principal reservoir and vector of TBEV are the hard ticks: *Ixodes ricinus*–a vector of TBEV-Eu and *Ixodes persulcatus*–a vector of TBEV-FE and TBEV-Sib. The course of infection with a particular subtype shows significant clinical differences with different case fatality rates in humans. The most likely route for humans to become infected with TBEV is a tick bite. However, TBEV can also be transmitted through the consumption of unpasteurized milk and milk products from infected animals such as goats, sheep, and cows [6].

The incidence of TBE has markedly increased during the past 20 years, which makes TBE, after Lyme disease, the second most serious disease transmitted by ticks [7]. TBEV is mainly endemic in Europe, Russia, and Asia [8,9]; however, the virus extends its range outside endemic areas. Recently, a new TBE endemic outbreak has been identified in Finnish Lapland, a place where TBEV has never been found before [10]. This suggests the expansion of the range of distribution of ticks and with them this threatening viral pathogen. Although vaccines against TBE based on inactivated viruses are available, the vaccination is not mandatory but only recommended for residents and tourists traveling to endemic areas. Vaccines are rarely used as a prevention tool, which results in more than 12,000 human cases reported annually [11,12]. Despite numerous strategies of research, currently there is no licensed therapeutic available for the treatment of TBEV infections [13]. Patients diagnosed with TBE infection are usually treated to alleviate the symptoms, e.g., to reduce the inflammation and intracranial pressure by anti-inflammatory drugs. Therefore, the development of new effective antiviral compounds is highly demanded. 

The TBEV envelope contains two viral proteins which play a major role in viral entry into the target cells: glycoprotein E and the small membrane protein prM/M. Protein E contains the major antigenic epitopes that induce the formation of protective antibodies during immune response. Both TBEV proteins possess at least one conserved *N*-glycosylation site [14]. The removal of glycans of viral proteins usually impairs their proper folding and stability. For TBEV, it has been proven that the loss of glycosylation of protein E affects the conformation of the protein, consequently reducing the infectivity of secreted virions [15]. It has been reported that the virus composed of glycoprotein E lacking the *N*-glycan chains was not infectious in a mouse model, confirming that glycosylation inhibition may be a new target for anti-TBEV compounds. 

The composition of glycan chains in viral glycoproteins can be modulated by glycosyltransferases (GTs) during the maturation step and thus may affect viral survival. Active compounds interacting with GTs could potentially contain covalently bound carbohydrate units. Previously, several uridine derivatives of various lipophilicity containing 2-deoxy sugar moieties were synthesized and some of them were tested for inhibitory activity against GTs [16,17,18,19]. Some of the compounds were reported to exert in vitro antiviral activity against viruses belonging to the Flaviviridae family (classical swine fever virus (CSFV) and HCV) as well as against the influenza virus from the Orthomyxoviridae family [16,20,21,22]. The in vitro efficacy of compounds against these viruses was related to the impaired maturation of viral proteins targeting the late step of *N*-glycosylation (cis- or very early medial-Golgi), confirming their wide spectrum of activity. Viruses belonging to the same family group exhibit a high degree of homology in terms of genomic organization, protein function, and replication strategy. As TBEV, together with HCV and CSFV, belongs to the Flaviviridae family, it can be therefore assumed that compounds having antiviral activity against HCV and CSFV may also have significant activity against TBEV. 

In the present study, the antiviral activity of uridine derivatives of 2-deoxy sugars was evaluated against TBEV. We showed that four compounds strongly inhibit the propagation of TBEV, exhibiting similar inhibitory profiles and low cytotoxicity to the host cells.

## 2. Results

### 2.1. Anti-TBEV Activity of Uridine Derivatives of 2-Deoxy Sugars

**Figure 1:**
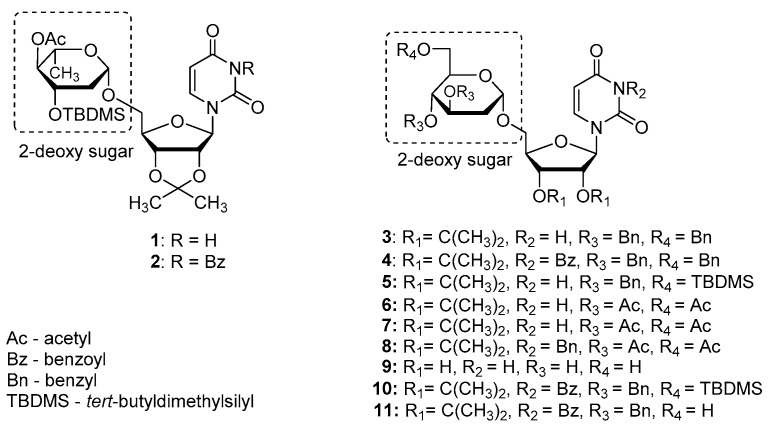
The structures of uridine derivatives of 2-deoxy sugars used in the study: **1**–**11**.

Previously, we synthesized uridine derivatives of 2-deoxy sugars (Figure 1) and reported that a few compounds from that series exert in vitro antiviral activity against CSFV [20], HCV [21], and the influenza virus [16,22]. In this comparative study, the synthesized compounds were evaluated for antiviral activity against another emerging virus—TBEV. To investigate the anti-TBEV activity, initially, the cytotoxicity of compounds in A549 cells was examined using the MTS assay to select nontoxic doses for further experiments. All tested compounds showed almost no cytotoxic effect at a concentration of 50 µM and the viability of A549 cells ranged from 95 to 100% for all tested compounds. The calculated cytotoxic concentration (CC_50_) values for compounds **1**–**11** (shown in Figure 1) required to reduce cell viability by 50% were 74, 141, 97, 119, 79, 196, 223, 225, 238, 121, and 141 µM, respectively. 

**Figure 2:**
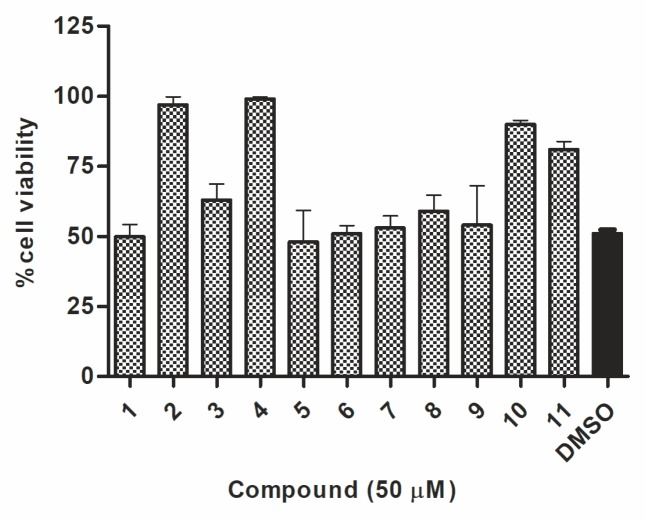
The effect of compounds **1**–**11** on the tick-borne encephalitis virus (TBEV)-induced cytopathic effect in A549 cells. A549 cells were infected with TBEV Hypr strain at a multiplicity of infection (MOI) of 0.1 and treated with 50 µM of compounds or DMSO (positive control). Cell death was measured and expressed as the percentage of cell viability 96 h post-infection (p.i.). Error bars indicate standard deviations from three experiments.

The preliminary screening of the 11 synthesized compounds was performed using Hypr and Neudoerfl TBEV strains in a cytopathic effect (CPE) inhibition assay and plaque reduction assay. All compounds were examined in the CPE inhibition assay, using the colorimetric assay to quantify the impact of synthesized compounds on cell death after infection. The TBEV Hypr strain (multiplicity of infection (MOI) of 0.1) without inhibitory treatment caused a severe CPE, including cell detachment and death detected 96 h post-infection (p.i.) in A549-infected cells. A protective effect of some compounds on cell survival indicates their potential antiviral activity. A high rate of A549 cell death (about 49%) was observed in Hypr-infected DMSO-treated cells (positive control) (Figure 2). The cells treated with uridine derivatives of 2-deoxy sugars (compounds **2**, **4**, **10**, and **11**) at 50 µM showed a low rate of cell death. Compounds **2** and **4** were the most active and completely inhibited the TBEV-induced CPE, protecting almost 99% of the A549 cells from cell death. Compounds **10** and **11** also decreased cell death and the calculated viability values after inhibitory treatment were 90 and 81%, respectively. The remaining tested compounds were not active, which was exhibited by a lack of inhibition of cell death after inhibitory treatment in comparison to the mock-treated Hypr-infected A549 cells. 

**Figure 3:**
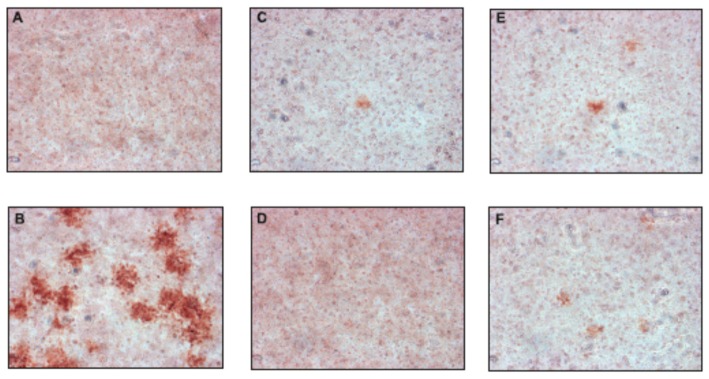
The effect of selected uridine derivatives of 2-deoxy sugars on TBEV propagation in A549 cells. A549 cells were infected with the TBEV Neudoerfl strain at an MOI of 0.001 (**B**–**F**) or mock infected (**A**). After virus removal at 2 h p.i., the cells were washed and overlaid with carboxymethylcellulose in Dulbecco’s Modified Eagle’s Medium with DMSO (**B**) or 50 µM of compounds **2** (**C**), **4** (**D**), **10** (**E**), and **11** (**F**). At 48 h p.i., cells were fixed and infected foci were visualized by immunostaining with the monoclonal anti-Flavivirus group antigen antibody (4G2).

Additionally, in the plaque reduction assay, the compounds were tested at a concentration of 50 µM for their ability to inhibit the propagation of TBEV at a low multiplicity of infection (MOI) of 0.001 to visualize single plaques using the immunoperoxidase monolayer assay (IPMA). Neudoerfl-infected A549 cells treated with DMSO were used as positive controls. The TBEV Neudoerfl strain is not a highly cytopathic virus, so after 48 h the areas of infected cells could be detected by the immunostaining of protein E. The results showed that compounds **2**, **4**, **10**, and **11** caused nearly a complete inhibition of TBEV infection in comparison to the positive controls (TBEV-infected cells treated with DMSO), which was manifested by the reduction of size and number of infected plaques after 48 h of inhibitory treatment (Figure 3). Compounds **1**, **3**, and **5**–**9** showed minor or no antiviral effect (data not shown). 

**Figure 4:**
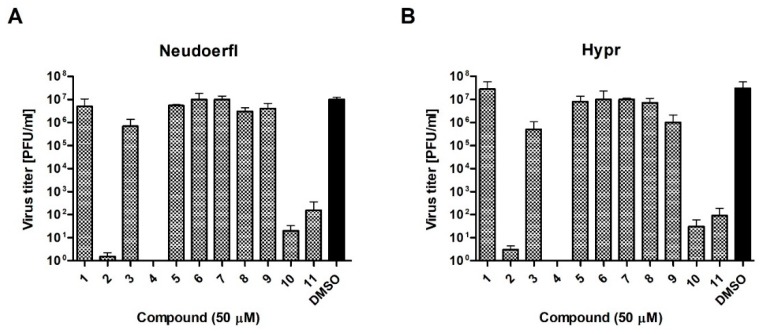
The effect of synthesized compounds on Neudoerfl and Hypr TBEV titers in A549 cells. A549 cells were infected with TBEV Neudoerfl (**A**) or Hypr (**B**) strain at an MOI of 0.1 and treated with 50 µM of compounds. At 72 h p.i., the supernatants were collected and viral titers were determined by the plaque assay. Error bars indicate standard deviations from three experiments.

To verify these preliminary results, the influence of compounds at a dose of 50 µM on TBEV growth was tested. Hypr and Neudoerfl TBEV titers from DMSO- and inhibitory-treated cells, infected at an MOI of 0.1, were determined in the culture supernatants at 72 h p.i. using a plaque assay. The obtained results confirmed that four of uridine derivatives—compounds **2**, **4**, **10**, and **11**- displayed an inhibitory effect on TBEV growth in A549 cells, which was in agreement with previous results (Figure 4). Nearly complete inhibition of viral growth was observed. Hypr and Neudoerfl strains without inhibitory treatment achieved titers of approximately 1 × 10^7^ plaque-forming unit/ml (PFU/ml) in A549 cells. All four active uridine derivatives significantly reduced viral titers compared to positive controls, indicating their antiviral activity. After treatment with compounds **2**, **4**, **10**, and **11**, the titers were reduced 1 × 10^7^–1 × 10^5^-fold compared to positive controls. Other compounds—**1**, **3**, and **5**–**9**-did not affect viral growth as no significant reduction in titers of both TBEV strains was observed. All these results strongly correlate with the results obtained in previous experiments (Figure 2 and Figure 3). 

### 2.2. Dose-Response of Anti-TBEV Activity of Uridine Derivatives of 2-Deoxy Sugars

**Figure 5:**
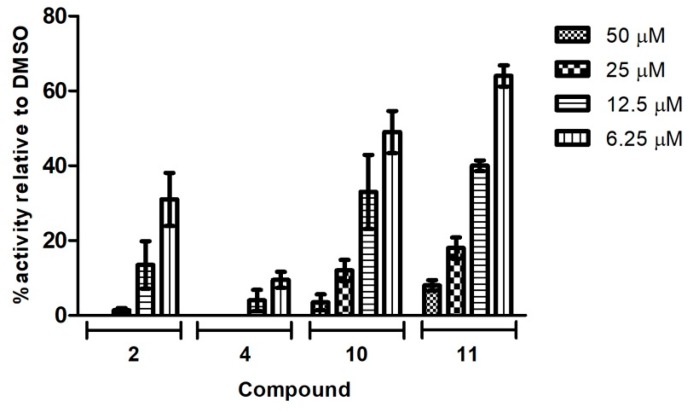
The effect of compounds on TBEV propagation in A549 cells. A549 cells were infected with the TBEV Neudoerfl strain at an MOI of 0.001. After virus removal at 2 h p.i., the cells were washed and overlaid with carboxymethylcellulose in D-MEM medium with DMSO or increasing doses of compounds. At 48 h p.i., cells were fixed and infected foci were visualized by immunostaining with the monoclonal anti-Flavivirus group antigen antibody (4G2). Plaques were counted and presented as a percentage in comparison to the number in DMSO-treated cells expressed as 100%. Error bars indicate standard deviations from three experiments.

The four compounds with the highest antiviral activity in the preliminary screening at 50 µM were further evaluated in dose-response assays. A549 cells infected with Neudoerfl TBEV strain (MOI = 0.001) were incubated for 2 days with an overlay medium containing increasing concentrations (0–50 µM) of compounds **2**, **4**, **10**, and **11**. The plaque reduction assay, which measures the extent of viral infection (visualization of the foci), was performed as described above. For all tested compounds, the dose-dependent reduction in average size and number of positive infected foci was observed in the plaque-reduction assay. As shown in Figure 5, compound **4** was the most active, exhibiting the lowest dose needed for the complete inhibition of plaque formation. After treatment with 50 and 25 µM of compound **4**, no plaques were detected. Additionally, the immunostaining of protein E revealed that compound **4**, at the lowest dose tested of 6.25 µM, significantly reduced the number of plaques by 90% in comparison to the TBEV-infected cells treated with DMSO. Moreover, no plaques were detected after treatment with 50 µM of compound **2**; 25 µM of this compound significantly reduced the number of plaques by 98%. Compounds **10** and **11** were slightly less active; 50 and 25 µM of these compounds reduced the number of plaques by around 82–96%. 

**Figure 6:**
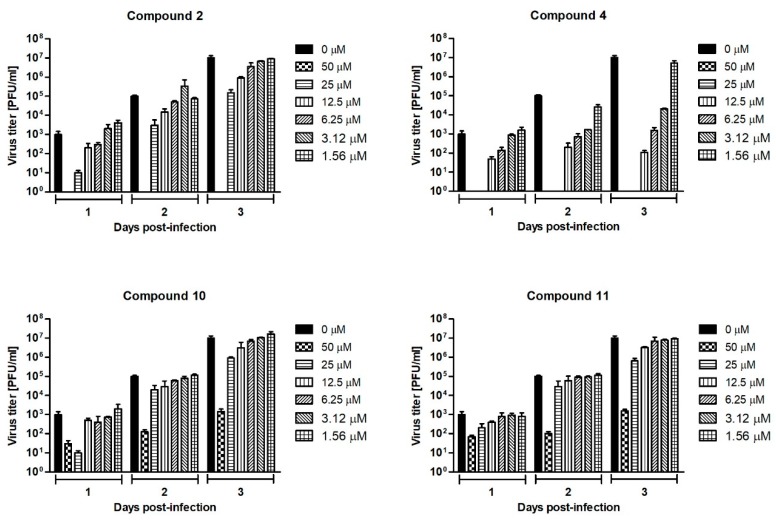
Dose-dependent effect of compounds **2**, **4**, **10**, and **11** on TBEV titers over a 3-day experiment. A549 cells were infected with TBEV Neudoerfl strain at an MOI of 0.1 and treated with different concentrations of compounds. Culture supernatants were collected at days 1, 2, and 3 post-infection and TBEV titers were determined by the plaque assay. Error bars indicate standard deviations from three experiments.

The culture supernatants collected on days 1, 2, and 3 p.i. from the Neudoerfl-infected A549 cells treated with compounds **2**, **4**, **10**, and **11** at concentrations ranging from 0 to 50 µM were subjected to the plaque assay to determine the TBEV titers. The highest titer (1 × 10^7^ PFU/ml) for positive control—the titer for TBEV collected from non-treated cells—was observed 3 days post infection. Therefore, the dose-response curves obtained from day 3 p.i. were used to calculate the half-maximum inhibitory concentration (IC_50_) value for each tested compound. All tested uridine derivatives of 2-deoxy sugars significantly reduced viral titers in a dose-dependent manner, indicating high anti-TBEV activity (Figure 6). The TBEV yields after inhibitory treatment were reduced each day post-infection, indicating their stable activity. Compound **4** was the most active because the decrease in viral titer observed 3 days p.i. was the highest. When compound **4** was added to the cells at concentrations of 50 and 25 µM, no virus was detected. At the lowest concentration of 6.25 µM, a 10^4^-fold reduction of the viral titer was observed in comparison to the TBEV-infected mock-treated cells. The calculated IC_50_ value for this compound was 1.4 µM. For compound **2**, treatment with a dose of 50 µM also reduced the TBEV titer to undetectable levels, confirming its activity. Other doses caused around 10^2^–10^1^-fold titer reduction. Compounds **10** and **11** at all tested doses caused 1 × 10^4^–1 × 10^1^-fold reduction in viral titers. The IC_50_ values for compounds **2**, **10**, and **11** were 5.3, 6.5, and 10.2 µM, respectively. The calculated selectivity indices (SIs) defined as the CC_50_/IC_50_ ratio for compounds **2**, **4**, **10**, and **11** were 26.6, 85, 18.6, and 13.8, respectively. 

### 2.3. The Effect of Uridine Derivatives of 2-Deoxy Sugars on Protein Synthesis

**Figure 7:**
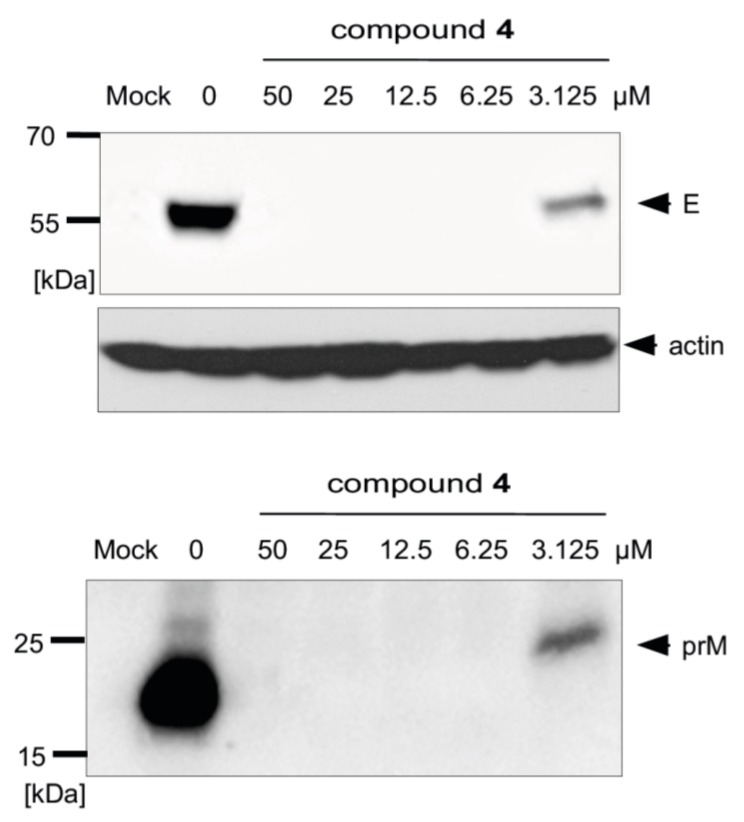
Effect of compound **4** on glycoprotein E and prM protein synthesis. A549 cells were infected with the TBEV Neudoerfl strain at an MOI of 0.1 and treated with different concentrations of the compound. At 48 h p.i., cells were lysed and proteins were separated by SDS-PAGE (gradient 10–20% polyacrylamide) under non-reducing conditions. Western blot analysis was performed using the monoclonal anti-Flavivirus group antigen antibody (4G2) for the detection of glycoprotein E, rabbit monospecific polyclonal serum for pr fragment detection, and anti-actin monoclonal antibody.

We previously proved that synthesized uridine derivatives of 2-deoxy sugars belong to *N*-glycosylation inhibitors [20]. The antiviral activity of studied compounds is based on the inhibition of the synthesis of viral proteins, which has a direct impact on virus propagation as has been shown for hepatitis C virus, classical swine fever virus, and influenza A virus. The antiviral effect was further confirmed by Western blot analysis, where the effect of compounds on TBEV glycoprotein synthesis was determined. For all tested compounds, a dose-dependent reduction in the yield of E and prM proteins in cells infected with the Neudoerfl TBEV strain was observed. The representative results for the most active compound, **4**, are shown in Figure 7. Compound **4** at a very low concentration (6.25 µM) completely inhibited the synthesis of both proteins, as they were not detected in the Western blot analysis. Both the non-glycosylated or under-glycosylated forms of both proteins were not detected in the experiment. This was probably due to the rapid degradation of incorrectly matured proteins, as was observed for other viruses in previous experiments. 

## 3. Discussion

Tick-borne encephalitis virus is as an important tick-borne pathogen which causes thousands of infections annually. A large percentage of infections are asymptomatic or have non-specific symptoms, thus many cases of infection are not reported by primary care physicians. This suggests that the scale of the problem is much greater. Inactivated virus-based vaccines which provide safe and reliable protection are available; however, they are offered only to at-risk travelers. Numerous studies have been carried out to develop new antiviral compounds against TBE virus. Although some compounds showed significant antiviral activity in in vitro studies, none of them were approved for use in humans.

Due to the urgent need for new effective anti-TBEV compounds, we evaluated the activity of previously reported compounds belonging to uridine derivatives of 2-deoxy sugars. In our previous studies, we reported that four compounds from these series exhibited antiviral activity against some members of the Flaviviridae family. These compounds were found to impair the maturation of viral proteins by interacting with glycosyltransferases active during the late steps (cis- or very early medial-Golgi) of the *N*-glycosylation process. Based on the high degree of homology between all members of the Flaviviridae family, e.g., CSFV, HCV, and TBEV, we expected that the activity of the selected compounds against TBEV would be similar to that of CSFV and HCV [20]. Additionally, we proved that uridine derivatives of 2-deoxy sugars possess antiviral activity against other viruses from outside the Flaviviridae family, e.g., the influenza virus belonging to the Orthomyxoviridae family, which indicates their broad spectrum of activity against many viruses [16,22]. 

The envelope glycoproteins, the most exposed structural elements of virions, play a vital role in the viral life cycle. They participate in the assembly of infectious particles and play a role in viral entry since they enable interaction with specific cell surface receptors and induce fusion between the viral envelope and the host cell membrane. Therefore, glycoproteins are assumed to be important factors of virulence and pathogenicity. TBEV possesses two viral membrane proteins, glycoprotein E and protein prM/M. Protein E mediates entry into the host cells and induces the generation of immune responses [23]. The major role of the prM/M membrane protein is a chaperone-like activity during the maturation of protein E [24]. Both TBEV envelope proteins are *N*-glycosylated. Glycoprotein E possesses one or two glycosylation sites depending on the viral strain and protein prM/M contains one conserved *N*-glycosylation site. The significance of the glycosylation process of viral proteins for viral growth, secretion, and pathogenicity of different types of viruses was confirmed in many studies. The important role of *N*-glycans attached to viral glycoproteins for TBEV infectivity has also been previously reported. Initially, it was shown that the inhibition of *N*-glycosylation reduced the secretion of progeny viruses from infected cells [25]. Moreover, targeting the *N*-glycosylation of protein E resulted in a significant decrease in the secretion of TBEV-like particles [24]. It has also been proven that *N*-glycosylation inhibition affects the folding and stability of protein E. The changes in the conformational structure of protein E affected by *N*-glycosylation inhibition results in reduced TBEV infectivity, which was confirmed in in vivo mouse studies [15]. These findings suggest that targeting the glycosylation process can be used to attenuate TBEV infection, which may be the basis for an innovative antiviral approach. 

In the current study, the antiviral activities of 11 uridine derivatives of 2-deoxy sugars were determined using two TBEV strains. The antiviral screening was performed with Neudoerfl and Hypr TBEV strains, which differ in virulence level. Initially, using the CPE inhibition assay as well as the plaque reduction assay in A549 cells, we tested the anti-TBEV activity of all compounds at 50 µM. Our results suggested that four out of the 11 compounds were active against TBEV, significantly inhibiting the viral infection, while compound **4** was the most active (Figure 2 and Figure 3). The results were further confirmed in other experiments where the activity of potential glycosylation inhibitors on viral growth was tested. We showed that, as in the previous experiments, only compounds **2**, **4**, **10**, and **11** strongly inhibited the growth of both TBEV strains, thus significantly reducing viral titers (Figure 4). Structural analysis of the studied compounds indicated that the presence of a benzoyl group at the uracil nitrogen had a positive impact on the antiviral activity, as was evidenced by the most active compounds **2**, **4**, **10**, and **11** containing a benzoyl group at the uracil nitrogen. 

Further, dose-dependent experiments were performed with the four selected compounds. The antiviral properties of the compounds at various concentrations were demonstrated in the plaque reduction assay as well as in the plaque assay to determine viral titers where the impact of different amounts of compounds was tested. All tested compounds caused a dose-dependent inhibition of TBEV production observed as the decrease of the average size and number of positive infected foci (Figure 5) and viral titer (Figure 6). TBEV was inhibited by these compounds at low micromolar concentrations. Compound **4** showed the highest antiviral activity, displaying an IC_50_ value of 1.4 µM. The IC_50_ values of the active compounds **2**, **10**, and **11** were 5.3, 6.5, and 10.2, respectively. The significant reduction in viral titer after inhibitory treatment was in agreement with previously published data, where a reduction in the virus yield of infectious recombinant virus expressing protein E lacking the *N*-glycans was observed [15]. 

As mentioned before, the inhibition of *N*-glycosylation also affects the folding and stability of the TBEV protein E. Therefore, the influence of selected uridine derivatives of 2-deoxy sugars on protein synthesis was examined. We demonstrated that compound **4** caused a dose-dependent decrease in the synthesis of proteins E and prM (Figure 7), which could be the main reason for the reduction of TBEV production in other experiments. Less glycosylated or non-glycosylated forms of proteins E and prM were not detected by Western blot, indicating the very quick degradation of incorrectly matured proteins. The same results were observed for proteins of other viruses, e.g., HCV, CSFV, and influenza virus, in our previous studies [16,20,21,22]. 

In conclusion, we demonstrated that compounds **2**, **4**, **10**, and **11** exert significant antiviral activity against TBEV. The observed results, together with our previous findings, confirm that the selected compounds possess a broad-range antiviral activity targeting the *N*-glycosylation process, and may constitute a novel class of inhibitors with a mechanism of action different from the currently tested antiviral drugs. These compounds may be the starting point for the synthesis of other antiviral compounds with some modifications to potentially improve their activity. 

## 4. Materials and Methods 

### 4.1. Antiviral Compounds, Cells, and Viruses

The compounds were synthesized as previously described [16,17,18]. The stock solutions of tested compounds were dissolved in dimethyl sulfoxide (DMSO) and stored at −20 °C until use.

A549 cells (adenocarcinomic human alveolar basal epithelial cells) (ATCC^®^ CCL-185™) were cultured in Dulbecco’s Modified Eagle’s Medium (D-MEM) (Sigma-Aldrich, Saint Louis, MO, USA) supplemented with 8% heat-inactivated fetal bovine serum (FBS), 2 mM l-glutamine, 0.2% bovine serum albumin, 25 mM HEPES buffer, 100 U/ml penicillin, and 100 µg/ml streptomycin at 37 °C under 5% CO_2_.

The Neudoerfl TBEV strain, kindly provided by Dr. Karin Stiasny (Center for Virology, Medical University of Vienna, Vienna, Austria), and the Hypr TBEV strain were used in this study. Both viruses were grown in A549 cells for 3–4 days until the cytopathic effect was visible. Virus titers were determined by the plaque assay. 

### 4.2. Cell Viability Assay 

A549 cell viability was measured by the CellTiter 96 AQ_ueous_ non-radioactive cell proliferation assay (MTS) (Promega, Madison, WI, USA) according to the manufacturer’s instructions. The half-maximal cytotoxic concentration (CC_50_) values of compounds that reduce cell viability by 50% was calculated using the GraphPad Prism software (version 5.01, GraphPad Software, San Diego, CA, USA) from the dose-response curves.

### 4.3. CPE Inhibition Assay 

A549 cells seeded in 96-well plates together with various doses of tested compounds or DMSO (positive control) were infected with the Hypr TBEV strain at an MOI of 0.1. After 96 h p.i., CPE (cell death) was determined using the colorimetric CytoTox 96^®^ Non-Radioactive Cytotoxicity Assay (Promega, Madison, WI, USA) according to the manufacturer’s instructions. This assay measures lactate dehydrogenase (LDH), a stable cytosolic enzyme that is released upon cell lysis. The absorbance at 450 nm was read using a plate reader.

### 4.4. Plaque Reduction Assay

Confluent monolayers of A549 cells in 12-well plates were inoculated with TBEV for 2 h at 37 °C. After the removal of the virus, the cells were washed and overlaid with carboxymethylcellulose in D-MEM medium with DMSO or different concentrations of compounds. Two days post-infection, cells were washed with phosphate-buffered saline (PBS), fixed with methanol, and infected foci were visualized by immunostaining with the monoclonal anti-Flavivirus group antigen antibody (4G2) (Absolute Antibody, Oxford, UK; diluted 1:1500 in PBS, 1% Tween 20, 5% FBS), followed with anti-mouse horseradish peroxidase (HRP)-conjugated secondary antibody (diluted 1:2000 in PBS containing 1% Tween 20 and 5% FBS). Plaques were detected using a Vector Nova Red kit (Vector Laboratories Ltd., Peterborough, UK) and counted. 

### 4.5. Plaque Assay 

Monolayers of A549 cells cultured in 24-well culture plates were inoculated with 10-fold dilutions of TBEV strains for 4 h at 37 °C. After the removal of the virus, the cells were overlaid with carboxymethylcellulose in D-MEM medium. After 5 days the medium was washed away and after a few washes with PBS, the cells were stained with naphthalene black to visualize the plaques. The virus titers were expressed as PFU per milliliter. 

### 4.6. Dose-Response of Anti-TBEV Activity of Uridine Derivatives of 2-Deoxy Sugars 

A549 cells were infected with the TBEV Neudoerfl strain (MOI = 0.1) and treated with tested compounds at a concentration range from 0 to 50 µM. Culture supernatants were collected on days 1, 2, and 3 post-infection and used for the determination of viral titers using the plaque assay. The dose-response curves prepared from data obtained at day 3 post-infection were used to calculate the half-maximum inhibitory concentration (IC_50_) values for each compound, indicating the concentration required to reduce the viral titer by 50% compared to the control, using GraphPad Prism software.

### 4.7. Western Blot Analysis 

Overnight, A549 cells in 12-well plates were infected with the Neudoerlf TBEV strain at an MOI of 0.1. After 2 h, the medium was collected and fresh medium with different concentrations of compounds or DMSO was added for 48 h. Cells were lysed at 4 °C for 1 h with TNET buffer (20 mM Tris–HCl (pH 7.4), 150 mM NaCl, 1 mM EDTA, 1% Triton X-100). Proteins were separated by SDS–PAGE under non-reducing conditions, transferred to PVDF membranes, and detected with a specific monoclonal anti-Flavivirus group antigen antibody (4G2) (1:1500 dilution), rabbit monospecific polyclonal serum raised against prM protein (1:1500 dilution), or anti-actin antibody (1:1000 dilution) as primary antibodies. Anti-rabbit or anti-mouse peroxidase (HRP)-conjugated secondary antibodies (diluted 1:2000) were used as secondary antibodies. Antigen–antibody complexes were detected using a Super Signal West Pico Substrate system (Pierce, Dallas, TX, USA) using Chemidoc XRSþ (BioRad, Hercules, CA, USA) and analyzed.
